# High Trait Rumination Is Associated with Blunted Nighttime Diastolic Blood Pressure Dipping

**DOI:** 10.1007/s12160-014-9617-8

**Published:** 2014-04-05

**Authors:** Jillian A. Johnson, Brenda L. Key, Faye S. Routledge, William Gerin, Tavis S. Campbell

**Affiliations:** 1 Department of Psychology, University of Calgary, 2500 University Dr NW, Calgary, Alberta T2N 1N4 Canada; 2 Anxiety Treatment and Research Centre and Mood Disorders Program, St. Joseph’s Healthcare, Hamilton, Ontario Canada; 3 Nell Hodgson Woodruff School of Nursing, Emory University, Atlanta, GA USA; 4 Department of Biobehavioral Health, The Pennsylvania State University, University Park, PA USA

**Keywords:** Ambulatory blood pressure, Blood pressure dipping, Nondipping, Trait rumination

## Abstract

**Background:**

Blunted blood pressure (BP) dipping during nighttime sleep has been associated with an increased risk of cardiovascular events. Psychological traits have been associated with prolonged cardiovascular activation and a lack of cardiovascular recovery. This activation may extend into nighttime sleep and reduce BP dipping.

**Purpose:**

This study aims to evaluate the association between trait rumination and nighttime BP dipping.

**Methods:**

Sixty women scoring either high or low on trait rumination underwent one 24-h ambulatory BP monitoring session. Self-reported wake and sleep times were used to calculate nighttime BP.

**Results:**

High trait rumination was associated with less diastolic blood pressure (DBP) dipping relative to low trait rumination. Awake ambulatory BP, asleep systolic blood pressure (SBP) and DBP, and asleep SBP dipping were not associated with trait rumination.

**Conclusions:**

In a sample of young women, high trait rumination was associated with less DBP dipping, suggesting that it may be associated with prolonged cardiovascular activation that extends into nighttime sleep, blunting BP dipping.

During nighttime sleep, blood pressure (BP) values decline, or “dip,” relative to daytime BP values [1]. A lack of nocturnal BP dipping, or nondipping, has been associated with target organ damage [2–4] and an increased risk of cardiovascular events in individuals with hypertension [5–8], along with cardiac hypertrophy and remodelling in a normotensive population [9]. These subclinical modifications in cardiovascular structure and function, such as increased left ventricular mass and carotid intima–media thickening, are considered a transitional stage in the development of cardiovascular disease and represent a strong predictor of cardiovascular risk [10].

Nondipping status has been associated with depression [11], obesity [12], sleep-disordered breathing [13, 14], and reduced physical activity [15], as well as demographic variables including African-American ethnicity [16] and female sex [17]. It is has also been suggested that nondippers may exhibit delayed cardiovascular recovery from stress-related elevations in BP that occur during the waking hours of the day [18]. Prolonged elevations in BP and heart rate (HR), or the total time that the cardiovascular system is activated following stress, have been suggested as relevant factors in the development of hypertension [19]. This association is apparent in various studies linking delayed cardiovascular recovery following stress to poor cardiovascular outcomes [20–23]. A recent meta-analysis of 41 studies examining the relationship between laboratory mental stress and cardiovascular outcomes reported associations between delayed cardiovascular recovery and greater carotid intima–media thickness and higher systolic blood pressure (SBP) and diastolic blood pressure (DBP) [24]. An association between prolonged recovery following daytime stress and nondipping may represent a potential pathway through which stress may be associated with hypertension and future cardiovascular risk.

An increasing body of research has identified psychological traits that characterize individuals who display prolonged cardiovascular recovery (i.e., return to pre-stress levels) following laboratory induced stress [25, 26]. Specifically, negative affect and depressed mood have been associated with sustained cardiovascular activation and slower recovery from stress [27–29]. More recently, rumination, defined as repetitive, unwanted thoughts focusing on problematic situations or events, the possible consequences of those events, and the accompanying emotions [30], has been identified as being of possible particular relevance for delayed cardiovascular recovery [31–33]. Rumination, often a symptom of depressed mood [34], has been identified as potentially relevant given that following a stressor, individuals may re-experience stress-related thoughts and emotions, leading to ongoing physiological arousal [26]. Specifically, the rumination arousal model suggests that rumination may cause or contribute to delayed cardiovascular recovery through perseveration of negative affect and associated autonomic arousal following a stressful encounter [32].

Preliminary support for the hypothesis that perseverative cognitive processes [35], such as worry or rumination, may impact cardiovascular activation and carry over from waking hours into sleep comes from a study of 24-h ambulatory HR and worry duration, a construct similar to rumination. In a sample of 52 healthy participants, worry duration was reported to be associated with higher ambulatory HR and lower ambulatory heart rate variability during both daytime and nighttime sleep [36]. While the assessment of circadian variation in ambulatory HR provides valuable preliminary evidence, ambulatory BP is more strongly prospectively related to hypertension [37] and would therefore provide more clinically meaningful information regarding the influence of rumination on pathogenic processes associated with the development of sustained high BP. In sum, rumination may be one mechanism through which stressors lead to delayed cardiovascular recovery as well as increased cardiovascular arousal manifested as nondipping during sleep.

The aim of the present study was to evaluate the association between trait rumination and nocturnal BP dipping, adjusting for daytime BP and sleep quality. Given the overlap that exists between symptoms of depressed mood and trait rumination, as well as previous reports of relationships between negative affect and BP dipping, the association between depressed mood and BP dipping was also assessed. It was hypothesized that relative to low trait rumination, high trait rumination would be associated with blunted BP dipping during nocturnal sleep.

## Method

### Participants

Participants were students recruited through the online research system at the University of Calgary. The sample pool was overwhelmingly female (>85 %), and all participants recruited for this study were women. Participants were categorized as either high (scoring above 530) or low (below 390) trait ruminators given their scores on the Stress Reactive Rumination Scale negative attributions and inferences subscale [38]. A total of 287 participants were screened, of which 60 (22 high and 38 low trait ruminators) were invited to participate. The cut points for categorizing high and low trait ruminators using the Stress Reactive Rumination Scale were determined based on a tercile split of scores collected from a sample of 104 undergraduate women at the University of Calgary in an unrelated pilot investigation [39]. Participants received course credit in exchange for their involvement in the study. Exclusion criteria included (a) use of antihypertensive medication or medications known to substantially affect BP; (b) previous diagnoses of diabetes mellitus, cardiovascular disease, uncorrected thyroid disease, history of alcohol or drug abuse (within the last 12 months), or renal or hepatic dysfunction; (c) inability to comply with the assessment procedures or inability to provide informed consent; and (d) pregnancy. All procedures and methods were approved by the University of Calgary Conjoint Faculties Research Ethics Board.

### Procedures

#### Screening Session

After providing informed consent, participants completed the Stress Reactive Rumination Scale, Beck Depression Inventory (BDI), demographic, and health-related questionnaires. Participants who met the inclusion criteria were invited to participate in the 24-h ambulatory BP assessment.

#### Ambulatory Assessment

Height and weight were measured and the participants were then instrumented with an oscillometric ambulatory BP monitor (see details below). BP was measured at 20-min intervals during the day and at 30-min intervals during the night. Ambulatory BP monitoring was conducted during a typical school day during which participants were instructed to follow their normal routine other than to abstain from vigorous physical activity. Immediately following each BP measurement while awake, participants recorded their posture, activity level, and the activity in which they were engaged in a paper-and-pencil ambulatory diary. The morning following nighttime BP collection, participants completed a sleep diary and sleep quality assessment. Ambulatory BP values were flagged as questionable and removed if (a) SBP values were <60 mmHg, (b) DBP values were <40 mmHg, (c) SBP values were 25 mmHg higher or lower than other values recorded during the same hour, (d) DBP values were 20 mmHg higher or lower than other values recorded during the same hour, or (e) the monitor generated a code indicating a possible source of artifact that would place the validity of the ambulatory BP reading in question. Mean SBP and DBP values were computed on the basis of all readings accepted as valid obtained during waking hours and during nighttime sleep as reported by each participant (missing/artifactual values were not replaced).

### Measures

#### Ambulatory BP Monitoring

Ambulatory BP was monitored over a 24-h period using the Oscar 2 oscillometric ambulatory BP monitor (Suntech Medical Instruments, Inc., Raleigh, NC, USA) at the time intervals described above. This monitor has been validated according to the International Protocol for the validation of BP measuring devices [40]. An appropriately sized BP cuff was selected according to the size of the participant’s arm and wrapped around the upper part of the nondominant arm.

#### Stress Reactive Rumination Scale

The Stress Reactive Rumination Scale negative attributions and inferences subscale is a nine-item self-rating questionnaire that provides a score between 0 and 900, with higher scores reflecting higher trait rumination [38]. The Stress Reactive Rumination Scale was adapted from the Response Styles Questionnaire-Ruminative Response Scale [41], and was designed to measure trait rumination in a college-aged sample and therefore contains instructions that may be particularly relevant for this population. Additionally, the scale was designed to measure trait rumination in a manner that is not confounded with depressive symptoms, a limitation of many other self-report rumination scales [38]. The internal consistency of the subscale, as analyzed by Cronbach *α*, was 0.89, whereas the retest reliability (*r*) was 0.71 (*p* < .001) [38].

#### Beck Depression Inventory (BDI)

The BDI [42] is a 21-item self-report questionnaire that assesses cognitive, behavioral, affective, and somatic symptoms of depression. Items are rated on a three-point Likert scale and summed to yield a total score that ranges from 0 to 63, with higher scores indicating more depressive symptomatology. Patients can be categorized as follows: minimal (total score <10), mild to moderate (total score 10–18), moderate to severe (total score 19–29), or severe (total score 30–63). It has been recommended that high scores on the BDI in samples of university students should not be interpreted as an indicator of depression as the BDI is highly correlated with other self report measures of psychopathology [43, 44]. This scale demonstrates internal consistency of *α* = 0.81 in non-psychiatric populations, and a test–retest reliability (*r*) greater than .60 [42].

#### Sleep Time and Quality

Participants were instructed to report time in bed, sleep onset latency, number of awakenings, wake time after sleep onset, and final wake time in a sleep diary upon awakening on the morning following the nighttime ambulatory BP monitoring. The use of individual self-reports of actual sleep and wake times, as opposed to arbitrary sleep/wake times pre-determined for the entire sample, may increase the validity of nighttime BP dipping values [45]. Due to concerns about the effects of sleep disruption associated with ambulatory BP monitoring on nighttime BP values [46], and that poor sleep quality is a strong predictor of nondipping profiles [47–49], a four-item measure was completed the morning after ambulatory BP monitoring to assess sleep quality on a five-point scale: “how difficult was it to fall asleep?”, “how difficult was it to stay asleep?”, “how would you rate your quality of sleep?”, “how did your sleep compare to most nights?”. The scores provided for these four questions were added together to obtain a total sleep quality score that ranged from 4 to 20, with lower scores indicating a relatively poor quality of sleep.

#### Body Mass Index (BMI)

Weights (to the nearest 0.1 kg) were obtained using a calibrated balance beam scale (Health O Meter 402KL ProSeries Doctor’s Scale, Sunbeam Products, Inc., Boca Raton, FL, USA) and height (to the nearest 1 mm) was measured using a fiberglass tape measure. Participants were barefoot and wore light clothing. These values were used to calculate BMI (kg/m^2^).

#### Smoking

The History and Current Status of Smoking questionnaire was used to assess the smoking status and characterize respondents as current smokers, former smokers, or non-smokers [50].

### Data Analysis

#### Comparison of Groups

To verify that the trait rumination groups were comparable on continuous demographic variables (i.e., age, BMI), as well as sleep measures (i.e., sleep onset latency, total sleep time, sleep quality) and BDI scores, a series of one-way (high vs. low trait rumination) between-subjects ANOVAs were conducted. To ensure that the trait rumination groups were comparable on the categorical demographic variables (i.e., smoking, ethnicity), Pearson chi-square tests (high vs. low trait rumination) were used.

#### Ambulatory BP Analysis

Nighttime BP was defined as all BP measures during “time asleep” until “time awoke” as self-reported. Dipping was defined as the difference (in mmHg) between mean daytime BP and mean nighttime BP. Simple correlations were conducted to examine the associations between SBP and DBP dipping, sleep variables, rumination scores, and BDI scores. To assess the association between trait rumination and nighttime BP dipping, one-way (high vs. low trait rumination) analysis of covariance (ANCOVAs) (adjusting for daytime SBP or DBP and sleep quality) were performed on SBP and DBP dipping. All data analyses were performed using SPSS for Windows Version 16.0 (SPSS Inc., Chicago, IL, USA). Statistical significance was set at *p* < .05 (two-tailed). All dependent variables were confirmed to be normally distributed.

## Results

### Participant Characteristics

Participants had a mean age of 22.2 (±5.7) years and a mean BMI of 22.4 kg/m^2^ (±2.6) (Table 1). A majority of the participants were White (66.7 %) (Table 1). A series of one-way ANOVAs indicated no significant differences between the high and low trait rumination groups on age, BMI, or any of the sleep variables (i.e., sleep onset latency, total sleep time, sleep quality), though they did differ on BDI scores (Table 1). Pearson chi-square analyses indicated no significant differences between the high and low trait rumination groups on ethnicity or smoking status (Table 1). Mean daytime, nighttime, and average 24-h BP values are presented in Table 2.

**Table 1:** Participant characteristics by trait rumination group

Demographic variable	Rumination			Statistical analyses	
	High ruminators	Low ruminators	Total	Results	
Age (years)	23.0 (6.5)	21.7 (5.2)	22.2 (5.7)	*F*(1,58) = 0.71	*p* = .404
BMI (kg/m^2^)	22.3 (2.7)	22.5 (2.7)	22.4 (2.6)	*F*(1,58) = 0.07	*p* = .796
Ethnicity				*χ* ^2^ (2, *N* = 60) = 2.40	*p* = .495
White	59.1 %	71.1 %	66.7 %		
Asian	27.3 %	13.2 %	18.3 %		
Other	13.6 %	15.7 %	15.0 %		
Smoking				*χ* ^2^ (2, *N* = 60) = 0.69	*p* = .710
Non-smoker	90.9 %	84.2 %	86.7 %		
Past smoker	4.5 %	10.5 %	8.3 %		
Current smoker	4.5 %	5.3 %	5.0 %		
Stress Reactive Rumination Subscale Score	611.0 (56.1)	284.3 (84.3)	404.1 (175.4)	*F*(1,58) = 261.93	*p* < .001
BDI total score	12.4 (8.2)	6.2 (6.4)	8.5 (7.7)	*F*(1,58) = 10.58	*p* = .002
Sleep onset latency (min)	19.0 (16.3)	17.4 (25.3)	18.0 (22.1)	*F*(1,53) = .06	*p* = .812
Total sleep time (min)	404.8 (92.8)	422.4 (73.3)	415.8 (80.8)	*F*(1,56) = .62	*p* = .434
Sleep quality	12.5 (3.7)	12.4 (3.2)	12.5 (3.4)	*F*(1,58) = .01	*p* = .926

Continuous variables are reported as mean (standard deviations); categorical variables are listed as column-wise percentages

*BDI* Beck Depression Inventory, *BMI* Body Mass Index

**Table 2:** Association between trait rumination and ambulatory blood pressure

	Rumination			ANOVA/ANCOVA^a^ results	
	High ruminators	Low ruminators	Total	*F*(*df*)	*p*
Awake SBP	124.9 (7.3)	127.1 (9.8)	126.3 (8.9)	*F*(1,58) = 0.83	.364
Awake DBP	75.0 (5.1)	75.7 (7.6)	75.4 (6.7)	*F*(1,58) = 0.15	.700
Asleep SBP	111.0 (10.3)	111.1 (8.2)	111.0 (8.9)	*F*(1,58) = 0.01	.983
Asleep DBP	62.1 (7.7)	59.7 (6.5)	60.6 (7.0)	*F*(1,58) = 1.66	.203
SBP dipping ^a^	13.9 (4.6)	16.1 (8.0)	15.3 (6.9)	*F*(1,56) = 0.73	.397
DBP dipping ^a^	12.9 (4.3)	16.0 (6.0)	14.8 (5.6)	*F*(1,56) = 4.62	.036

Variables reported as mean (SD); dipping was calculated as a change score (awake − asleep)

*SBP* systolic blood pressure, *DBP* diastolic blood pressure

^a^Mean awake blood pressure and sleep quality used as covariates

### Ambulatory BP Monitoring

After editing, a mean of 83.3 % of expected daytime and nighttime ambulatory BP measurements were retained for statistical analysis. All participants in the study had at least 18 pairs of SBP and DBP readings during waking hours and at least 9 pairs during nighttime sleep. There were no significant associations between SBP dipping and sleep onset latency (*r* = −.047, *p* = .735), total sleep time (*r* = −.035, *p* = .796), sleep quality (*r* = .050, *p* = .703), or the item “how did your sleep compare to most nights?” (*r* = .013, *p* = .925). Similarly, DBP dipping was not associated with sleep onset latency (*r* = .024, *p* = .862), total sleep time (*r* = −.038, *p* = .783), sleep quality (*r* = .111, *p* = .397), or the item “how did your sleep compare to most nights?” (*r* = .122, *p* = .366). Simple correlations revealed no significant associations between BDI scores and SBP dipping (*r* = −.216, *p* = .097) or DBP dipping (*r* = −.249, *p* = .055). Trait rumination was not significantly correlated with sleep onset latency (*r* = .112, *p* = .414), total sleep time (*r* = −.122, *p* = .369), sleep quality (*r* = .050, *p* = .704), or the item “how did your sleep compare to most nights?” (*r* = .078, *p* = .564).

Results of the one-way ANOVAs revealed no significant associations between trait rumination and mean awake or asleep ambulatory BP levels (Table 2). One-way ANCOVAs of nighttime DBP dipping (co-varying daytime DBP and sleep quality) indicated that high trait ruminators had less DBP dipping than low trait ruminators, *F*(1,56) = 4.62, *p* = .036, partial *η* ^2^ = .076. Similar analyses of SBP dipping revealed no significant association between trait rumination and SBP dipping, *F*(1, 56) = 0.73, *p* = .397, partial *η* ^2^ = .013 (Table 2). Upon inclusion of BDI scores as an additional covariate in the above analyses, the association between DBP dipping and trait rumination was no longer significant, *F*(1,55) = 2.97, *p* = .091, partial *η* ^2^ = .051, and the association between SBP dipping and trait rumination remained nonsignificant, *F*(1,55) = .34, *p* = .561, partial *η* ^2^ = .006.

Semi-partial correlations were examined to assess the unique associations between psychological variables (trait rumination, BDI score) and DBP dipping. For trait rumination and DBP dipping, the difference between the zero-order correlation (*r* = −.269, *p* = .037) and the semi-partial correlation (*pr* = −.203, *p* = .091) suggests that that only 0.4 % of the 7.2 % variance between trait rumination and DBP dipping is accounted for by sleep quality, average daytime DBP, and BDI scores. For BDI scores and DBP dipping, the difference between the zero-order correlation (*r* = −.249, *p* = .055) and the semi-partial correlation (*pr* = −.073, *p* = .386) suggests that that 3.1 % of the 6.2 % variance between BDI scores and DBP dipping is accounted for by sleep quality, average daytime DBP, and trait rumination.

## Discussion

This study examined the impact of high and low levels of trait rumination on BP dipping status during nighttime sleep. The central finding was that high trait ruminators exhibited less DBP dipping during nighttime sleep compared to low trait ruminators despite statistical adjustments for self-reported sleep quality and average awake DBP. The inclusion of depression scores only slightly attenuated this effect, suggesting that the association between rumination and DBP dipping is not substantially explained by depressed affect in this sample. Previous research has demonstrated the role of trait rumination on BP recovery from mental stress in the laboratory (e.g., 31,32) and on sleep quality [51]; however, this is the first study to suggest that BP profiles during sleep may be influenced by delayed cardiovascular recovery associated with higher levels of trait rumination. The findings of this study add to our understanding of the factors that may play a role in BP dipping profiles by suggesting that certain psychological traits, such as rumination, may serve to prolong cardiovascular activation into periods in which stressors are absent.

In the present study, only DBP dipping, and not SBP dipping, was associated with trait rumination. Previous research assessing associations between trait rumination and cardiovascular recovery from mental stress in the laboratory have reported similar outcomes [31, 32], with DBP recovery and not SBP recovery associated with trait rumination. This discrepancy may be due to differences in the hemodynamic regulation of SBP and DBP among high versus low trait ruminators. Given that peripheral resistance is a stronger contributor to DBP than SBP [52], high trait ruminators may display this pattern of autonomic specificity relative to low trait ruminators, contributing to a blunted nighttime DBP dipping profile. DBP is considered to be reflective of the flexibility of artery walls and has been reported as a stronger predictor of coronary heart disease risk than both SBP and pulse pressure in a sample of people less than 50 years of age [53], and therefore may have increased prognostic significance in younger individuals. In light of the reported associations between rumination and both DBP recovery and DBP dipping, future research would benefit from elucidating the hemodynamic underpinnings of this pattern of results.

Similar to previous reports of prolonged BP recovery to laboratory mental stress characteristic of rumination [26, 31, 32], the tendency for relatively blunted BP dipping among high trait ruminators in the present study may represent diminished recovery from daily stress during nighttime sleep. These findings add to existing evidence that perseverative thoughts or ruminative cognition during the day may be associated with prolonged physiological recovery during sleep. In an ambulatory study, Brosschot and colleagues [36] noted reduced nighttime parasympathetic tone (as indexed by heart rate variability) and higher heart rate among participants who had reported experiencing a greater number of stressors in the preceding day relative to those who reported experiencing less stress. Further, Hall and colleagues [54] reported decreased parasympathetic and increased sympathetic function during a period of nighttime sleep (verified by polysomnography) preceding a public speaking stressor. Brosschot and colleague’s theory of perseverative cognition [55] suggests the possibility of unconscious perseverative or ruminative thought occurring during periods of sleep that might account in part for such findings, although this remains speculative in the absence of evidence.

Similar to the concept of cardiovascular recovery, McEwen [19] describes a type of allostatic load that is presented as an inability to shut off allostatic responses after a stressor is terminated. Over time, these responses may create a sustained burden on the allostatic system (i.e., the cardiovascular system), leading to damage (i.e., cardiovascular disease). The present study advances McEwen’s theory by suggesting that this type of allostatic load may also describe the phenomenon of blunted BP dipping during nighttime sleep in those with high trait rumination. Specifically, having high levels of a personality trait such as rumination may lead to sustained allostatic responses such as DBP during sleep, preventing the normal dipping pattern, and potentially increasing cardiovascular risk.

### Limitations

While both delayed BP recovery from mental stress [36] and poor BP dipping [56] have been associated with autonomic dysregulation, the cross-sectional nature of the study design precludes the ability to make definitive causal statements. Second, the mean BP dipping value for both high and low trait ruminators in this study was within the normal range (>10 % decrease during nighttime sleep), indicating that the sample may not be classified as conventional “nondippers.” It is important to note, however, that continuous measures of BP dipping may be more reliable and provide greater predictive utility relative to an arbitrary threshold of risk [45, 57, 58]. For every 5 % decrease in BP dipping, it has been estimated that there is a 20 to 30 % rise in the risk of cardiovascular death [8]. Third, BP dipping was assessed on only one occasion. Concerns have been raised about the reproducibility of dipping data based on one 24-h period [46, 56–58]. Our approach to addressing this potential difficulty was to consider BP dipping as a continuous rather than categorical value [59], use individual self-reported sleep and wake times to determine the nighttime period (to calculate dipping), and to collect data during a standardized period (i.e., over the course of a routine school day). These measurements are consistent with recommendations regarding the assessment of ambulatory BP during a single session [45, 58] and minimize potential issues regarding reliability and reproducibility. Fourth, although there is literature suggesting that an increased tendency to ruminate is reliably associated with greater habitual rumination [60], this study did not measure the frequency or duration of ruminative episodes during the ambulatory monitoring period. Similarly, while the association between rumination and BP recovery during nighttime sleep (i.e., dipping) supports our hypothesis and extends previous findings of associations between rumination and delayed BP recovery in the lab in response to mental stress, a thorough assessment of negative affect (e.g., depression, anxiety, anger, etc.) was not conducted. Given that there are features of rumination that overlap with other negative affect traits, the study would have benefited from a more thorough characterization of psychological states and traits to verify the specificity of this particular trait. Finally, it should be noted that by categorizing women as high and low trait ruminators using a tercile split of Stress Reactive Rumination Scale scores, this introduced potential for regression toward the mean during the subsequent ambulatory BP monitoring.

### Future Directions

Given previously reported associations between sleep quality and both trait rumination [51] and ambulatory BP monitoring [47–49], it is possible that trait rumination might impact BP dipping through sleep disruption associated with activation of an ambulatory BP monitor. In the present study, our pencil and paper measure of sleep quality that included an item specifically asking how participants’ sleep compared to most nights was not associated with either trait rumination or BP dipping. Future research evaluating the impact of psychological traits on BP dipping would benefit from using an objective measure of sleep quality, such as wrist actigraphy. Further, the inclusion of laboratory-based reactivity/recovery data from mental stress together with ambulatory monitoring would provide a more complete representation of the recovery patterns associated with trait rumination. Finally, the study sample was comprised of young, healthy, normotensive women free from antihypertensive medications and cardiovascular disease. Characterizing the magnitude and clinical implications of blunted BP dipping associated with trait rumination in hypertensive patients or those at increased risk of cardiovascular disease seems warranted.

In summary, trait rumination is associated with less DBP dipping during nocturnal sleep in a sample of healthy young women. These results add to the growing body of knowledge suggesting that rumination may play a role in preventing cardiovascular recovery, including recovery defined as reductions in BP during nighttime sleep.
